# Colorectal cancer stem cells properties and features: evidence of interleukin-8 involvement

**DOI:** 10.20517/cdr.2019.56

**Published:** 2019-12-19

**Authors:** Fabiana Conciatori, Chiara Bazzichetto, Italia Falcone, Gianluigi Ferretti, Francesco Cognetti, Michele Milella, Ludovica Ciuffreda

**Affiliations:** ^1^Medical Oncology 1, IRCCS - Regina Elena National Cancer Institute, Rome 00144, Italy.; ^2^Section of Oncology, Department of Medicine, University of Verona School of Medicine and Verona University Hospital Trust, Verona 37126, Italy.; ^3^SAFU, Department of Research, Advanced Diagnostics, and Technological Innovation, IRCCS - Regina Elena National Cancer Institute, Rome 00144, Italy.

**Keywords:** Interleukin-8, colorectal cancer, cancer stem cells, tumor microenvironment

## Abstract

Colorectal cancer (CRC) still remains a disease with high percentage of death, principally due to therapy resistance and metastasis. During the time the hypothesis has been reinforced that CRC stem cells (CRCSC) are involved in allowing intratumoral heterogeneity, drug escape mechanisms and secondary tumors. CRCSC are characterized by specific surface markers (i.e., CD44 and CD133), signaling pathways activation (i.e., Wnt and Notch) and gene expression (i.e., Oct4 and Snail), which confer to CRCSC self-renewal abilities and pluripotent capacity. Interleukin (IL)-8 is correlated to CRC progression, development of liver metastases and chemoresistance; moreover, IL-8 modulates not only stemness maintenance but also stemness promotion, such as epithelial-mesenchymal transition. This review wants to give a brief and up-to-date overview on IL-8 implication in CRCSC cues.

## Introduction

Colorectal cancer (CRC) is one of the highest incidence tumor worldwide, with around 10% of 5-year relative survival rate for both metastatic rectal and colon cancer. Despite prevention, early diagnosis and personalized medicine era significantly improved the response rate to conventional treatments, therapy resistance and metastasis are still the main causes of CRC death^[1]^. During the time accumulating evidence suggested that CRC stem cells (CRCSC) are one of the key actors involved in drug resistance and metastatic spread. Indeed, cancer stem cells (CSC) are characterized by the regulation of specific signaling cascades, such as Wnt, transforming growth factor (TGF)-β and Notch pathways, self-renewal and clonal repopulation capabilities, which are all influenced by the surrounding microenvironment^[2]^.

CSC are able to undergo to infinite cell cycle division and through asymmetric division and their pluripotent capacity, they do not only display self-renewal abilities but they can also produce a plethora of heterogeneous cancer cells: these cancer cells display plasticity properties to better adapt in new microenvironment and spread metastases in different tissues and organs^[3]^. Novel therapeutic drugs with the aim of killing CSC are currently developing. More specifically, therapies against CSC include targeting of: surface markers, ATP-driven pumps, key signaling cascades such as Notch, Hedgehog and Wnt pathways, and tumor microenvironment (TME) elements^[4]^. Indeed, similarly to the well-known role of tumor-stroma interactions (TSI) in cancer progression, it is established that physical interactions and soluble factors in microenvironment work in concert to balance self-renewal and differentiation stimuli of both normal and CSC^[5]^.

Both normal and tumor niches are characterized by extracellular matrix, soluble factors (cytokines), different immune cells, endothelial cells and fibroblasts^[6]^. Homeostasis of intestinal epithelium is maintained by normal intestinal stem cells (ISCs) in the niche at the base of the intestinal crypt, where two subpopulations of ISC, Leucine-rich-repeat-containing G-protein-coupled receptor5 (Lgr5)^+^ ISCs and +4 ISCs, are able to differentiate in intestinal epithelium^[7]^. Among stem cells, mesenchymal stem cells (MSC) are multipotent stromal cells and represent a key cell population in CSC niche through cytokines production: indeed, MSC release CXCL12, interleukin (IL)-6, and IL-8 which bind their membrane receptor(s) on CSC surface, thus activating NF-κB pathway. NF-κB regulates migration and invasion of CSC, production of soluble factors in the tumoral niche and the epithelial-mesenchymal transition (EMT) inducers Slug, Snail and Twist^[8,9]^. EMT represents a critical step during metastasis and cancer progression, as differentiated epithelial cells convert to motile mesenchymal cells. Tumor angiogenesis and hypoxic conditions also display a pivotal role in CSC microenvironment: in particular, the main actor of angiogenic mechanism vascular endothelial growth factor (VEGF), activated as hypoxia-inducible transcription factor target gene, increases proliferation, self-renewal and tumorigenicity of CSC^[8]^.

IL-8 is proinflammatory CXC ELR (Glu-Leu-Arg)+ chemokine, mainly known for its implication in neutrophil chemoattraction. Compelling evidence has revealed that IL-8 is also involved in CRC progression, development of liver metastases and chemoresistance, by affecting not only TSI, but also CSC features and properties, such as CSC generation and maintenance, EMT and tumor angiogenesis^[10]^. IL-8 displays its biological functions, through its binding to the heterotrimeric G protein-coupled receptors, CXCR1 and CXCR2, expressed by monocytes, as well as endothelial, tumor and stromal cells^[10,11]^. During the last years, specific CXCR1/2 antagonists and IL-8 neutralizing antibodies were developed and their use in combination with chemotherapeutic and molecular target inhibitors represents a new promising strategy for cancer therapy. For example, treatment with CXCR2 antagonist, SCH-527123, increases sensitivity of CRC cells to chemotherapeutic agents^[12]^.

In this review, we will briefly describe CRCSC markers and features, highlighting the role of microenvironment and IL-8 in affecting stemness properties.

## General features of CRCSC

CRCSC are heterogeneous cells and their classification occurs also for molecular and functional features about self-renewal, pluripotent and plasticity capabilities. Moreover, not all the CSC in primary tumors display migration abilities: indeed, Brabletz *et al*.^[13]^ discriminated stationary CSC (SCSC) from migrating CSC (MCSC). In the epithelial tissue, SCSC are actively involved in each step of tumor progression. As opposite, MCSC, which have undergone EMT thereby displaying high levels of nuclear β-catenin, possess motility traits and are able to spread in other tissue to form metastatic tumor mass. Very interestingly, specific MCSC can lead to organ-specific metastasis, according to their markers. For example, it has been reported that the cell surface markers, CD110 and CDCP1, are displayed by MCSC which metastasize to liver and lung, respectively^[14]^.

As reported above, in addition to their multi-lineage differentiation potential, CSC display asymmetric division, by which they can renew their selves. Even if several canonical pathways are involved in self-renewal maintenance (i.e., Notch and Wnt), O’Brien and her colleagues demonstrated that the inhibitor of DNA binding (ID) proteins, a family of homologous helix-loop-helix transcriptional regulatory factors, are specifically involved in self-renewal property^[15]^. Indeed, the authors showed that ID1 and ID3 promote self-renewal through p-21 and they are involved in oxaliplatin-resistance of CSC, in CRC cell lines and xenografts^[16]^. Two years later, the same scientists revealed that the downregulation of Bmi-1 reduces self-renewal capability of CRCSC, thereby highlighting new actor(s) in stemness regulation^[17]^.

Wnt/β-catenin signaling is an evolutionarily conserved pathway, mainly involved in embryogenesis, tissue homeostasis and stemness. Signaling through this pathway occurs via a finely-regulated balance between accumulation and degradation of β-catenin, which displays its main function in the nucleus, where it promotes Wnt target oncogenes such as cyclin D1 and c-myc. As Wnt growth factor (GF) binds its specific receptor Frizzled, β-catenin destruction complex [composed by glycogen synthase kinase-3β, adenomatous polyposis coli (APC), CK1 and Axin] is recruited to the membrane, thus leading to β-catenin phosphorylation and ubiquitination blockade^[18]^. Mutations in *APC* gene, which occur in about 50% of CRC, mainly co-occur with additional genetic mutations in KRAS, Notch, or phosphoinositide 3-Kinase (PI3K) cascade, thus hyperactivating β-catenin signaling and contributing to CSC properties^[2]^.

In CRCSC, Notch signaling activation levels are elevated, due its role in inhibiting apoptosis and maintaining an undifferentiated state. Delta-like (DLL)1, DLL3, DLL4, JAGged (JAG)1 and JAG2 with a delta-serrate-lag 2 domain are the main ligands of Notch1, Notch2, Notch3 or Notch4 receptors: the binding activates Notch intracellular domain, which acts in the nucleus as a promoter of transcription factors for stemness genes and NF-κB^[15]^. The TGF-β family is also involved in CRCSC features and comprises over 40 members: the binding to the membrane receptors leads to the activation of the intracellular receptor-regulated SMAD, which acts as a transcription factor of stemness EMT genes, such as Snail and IL-8^[15]^.

During cancer progression, stochastic genetic and epigenetic mutations envisage tumor heterogeneity and CSC self-renewal; however, it is now well-established that CSC plasticity is driven also by microenvironmental stimuli and variations. Indeed, TME elements cause stemness characteristics acquisition by cancer cells, mainly via EMT induction; in that respect, much evidence highlights the key role of cytokines in cell-reprogramming into CSC^[19]^.

## Markers of CRCSC

CSC represent a phenotypic subset of cancer cells, characterized by specific surface and intercellular markers, many shared between blood and solid tumors. These molecules display cellular biological functions, which could in turn impact cancer progression: thus, their identification and study are crucial to improve therapeutic approaches. The expression of these markers is not a specific tumor property, but represents a CSC dynamic feature, which is modulated in quantity and phenotype during cancer progression. Nevertheless, CSC can be characterized and isolated by assessing the co-expression of different markers and not by the presence of just one of them^[20]^. Here, we summarize the main CRCSC markers.

### CD133

CRCSC were first isolated according to CD133 surface expression. CD133, also called Prominin-1, is a transmembrane glycoprotein and specifically localized in membrane protrusions, such as intestinal microvilli; several evidence showed that CD133 is involved in cellular self-renewal, tumorigenesis and metastasis^[21]^. Indeed, O’Brien *et al*.^[22]^ demonstrated that in immunodeficient mice, human colon cancer-initiating cells are CD133^+^ and as opposite, CD133^-^ cells are unable to initiate tumor growth. Similar results were shown also in a paper by Ricci-Vitiani *et al*.^[23]^: they demonstrated that undifferentiated tumorigenic CD133^+^ cells cause CRC and these cells should be investigated for further therapies. Recently, it has been shown that CD133 expression correlates with the degree of tumor differentiation and size in a clinical series of CRC^[24]^. However, even if the role of CD133^+^ cancer cells in tumor initiation seems to be established, other opposite data were reported: for example, it has been shown that CD133^-^ cells represent the most aggressive cell populations during metastasis, thereby hypothesizing a controversial role for CD133 in CRCSC^[25]^.

### CD44

The binding of the transmembrane glycoprotein CD44 to its ligand hyaluronic acid is responsible for cell-to-cell contact, cell-matrix interactions, cell adhesion and migration; through the RNA alternative splicing of ten intermediate exons, CD44 has more than 20 isoforms, 12 of which are the most common. It has been demonstrated that the expression of the isoform CD44v2 upregulates xenografts tumor initiation, whereas CD44v6 expression is involved in metastases formation in xenografted mice^[26,27]^. Furthermore, several data correlate the expression of CD44v6 and poor prognosis of CRC patients: indeed, Saito *et al*.^[28]^ demonstrated that high level of CD44v6 expression is an independent poor prognostic factor in disease-free survival (DFS) and overall survival (OS); moreover, the CD44v6 expression is higher than CD44 in stage II and III sporadic CRC, thus confirming that CD44v6 is a more useful biomarker as compared to CD44^[29]^. Furthermore, a number of studies revealed a correlation between CD44 and CD133 expression: for example, CD44 and CD133 could be used as biomarkers for hepatic metastases of CRC, due their mRNA co-expression in liver metastases^[30]^. As opposite, CD44^+^/CD133^-^ subpopulation displays the strongest invasion and migration capability in *in vitro* CRC cell models, thus highlighting that these markers correlations and their biological implications in cancer still require further studies^[31]^.

### CD166

CD166, also known as activated leukocyte cell adhesion molecule, is a type 1 transmembrane glycoprotein, which belongs to the immunoglobulin superfamily, and is mainly expressed by a restricted subset of cells with motility properties. According to such stem property of migration, Levin *et al*.^[32]^ demonstrated that undifferentiated cells at the base of the intestinal crypt display higher levels of surface CD166 as compared to differentiate cells. By affecting cell-to-cell contact, CD166 is involved in development and maintenance of tissue organization^[33]^. In 2007, Dalerba *et al*.^[34]^ identified CD166 as an additional marker of CRCSC membrane of Epithelial Cell Adhesion Molecule (EpCAM)^high^/CD44^+^ cells. Although the physiological role of CD166 in intestinal cells is still poorly known, its implication in cancer progression and metastasis is well recognized, as well as its potential as a therapeutic target in CRC. In that respect, Tachezy *et al*.^[35]^ showed that CD166 is significantly down-expressed in metastases as compared to primary tumors and its presence is a positive prognostic factor for OS.

### EpCAM

As previously mentioned, EpCAM, also known as Epithelial-Specific Antigen or CD326, is another CSC marker, expressed in 85% of colorectal carcinomas^[36]^. EpCAM is a transmembrane glycoprotein involved in epithelial cells adhesion, through homophilic and heterophilic interactions and represents a marker for circulating tumor cells^[37]^. As a cell adhesion molecule, it is not surprising that EpCAM expression favors cell motility and cell migration, by promoting specific mechanisms, such as EMT^[38]^. Moreover, Lin *et al*.^[39]^ showed that EpCAM is involved in affecting CSC features, such as self-renewal, growth, and tumor-initiating abilities: indeed, they demonstrated that deregulation of EpCAM represses the expression of reprogramming genes, i.e., c-Myc, Oct4, Nanog, and Sox2. Very recently, Leng *et al*.^[40]^ demonstrated that cell subpopulation, which concurrently expresses CD44^+^, EpCAM^+^ and Lgr5^+^, displays self-renewal capacity in preclinical models of CRC.

### Lgr5

In 2007, Lgr5, also known as GPR49, was identified as a marker of both colon normal stem cells and CSC^[41]^. Lgr5 is a seven-transmembrane G-protein-coupled receptor and represents one of the main targets of Wnt signaling^[42]^. Moreover, a feedback loop leads to the association of Lgr5 to Frizzled/Lrp Wnt receptor complex, thereby enhancing Wnt signaling^[43]^. Lgr5 is involved in maintenance of critical features of CSC, such as self-renewal, by upregulating stemness genes (i.e., Oct4, Sox2, c-Myc, and KLF4) as compared to Lgr5-negative cells^[40]^. The involvement of Lgr5 upregulation in all the phases of cancer transformation is now well recognized: indeed, its overexpression begins at the early stage of colorectal tumorigenesis and remains up to late events^[44]^. Furthermore, the analysis of Lgr5 expression in 296 CRC patients, treated with the chemotherapeutic agent 5-fluorouracil (5-Fu), revealed that high Lgr5 protein levels significantly correlate with advanced stages and shorter DFS^[45]^. All this evidence suggests that Lgr5 expression could be a good prognostic factor and potential target in CRC.

### ALDH1

Despite data about their role in CRC are still controversial and need for further investigations, aldehyde dehydrogenase 1 (ALDH1) and its several isoforms represent new stem markers. ALDH1 is an oxidoreductase enzyme that oxidizes intracellular aldehydes and protects stem cells through oxidative stress, thereby increasing longevity of both normal and CSC and interfering with several chemotherapeutic agents^[46]^. Indeed, ALDH1A3 isoform contributes to chemoresistance, as confirmed by its silencing which causes increased chemosensitivity, in preclinical models of CRC cells^[47]^. However, Hessman *et al*.^[48]^ demonstrated that ALDH1 is expressed in non-metastatic CRC, thereby suggesting its role and potential involvement as a druggable target only in early phase of cancer progression.

### Dclk1

Markers of normal and CSC are shared and this represents a drawback in development of cancer drugs directed against stem cells: as opposite, doublecortin like kinase 1 (Dclk1) is a specific CSC marker and does not mark normal stem cells^[49]^. Dclk1 is a microtubule-associated serine-threonine protein kinase involved in tumor stemness and progression, by promoting survival signaling, migration and tumor cell pluripotency, mainly through an intensive crosstalk with miRNAs^[50,51]^. It has been shown that Dclk1 is able to inhibit caspases gene expression, thereby blocking apoptosis pathway and increasing resistance to 5-Fu treatment in *in vitro* CRC models^[52]^.

## Role of cytokines in stemness

Both tumor and stromal cells release cytokines, classified in GFs, chemokines, angiogenic factors, and interferons, all soluble factors involved in TSI and drug resistance/sensitivity^[10]^. Through the binding to specific membrane receptors, they promote the activation of signaling pathways involved in promoting target cells stemness: in this way, CRCSC plasticity includes a balance shift between stem and non-stem state.

In Table 1, we summarize some examples of TME cells which regulate stemness through the production of specific cytokines and soluble factors. As highlighted above, Wnt/β-catenin is one of most relevant signaling cascade involved in stemness, thus it is not unexpected that several cytokines receptors cross-talk and activate the Wnt pathway. For example, Vermeulen *et al*.^[53]^ demonstrated that myofibroblasts-secreted hepatocyte growth factor (HGF) induces β-catenin stability, through the binding to HGF receptor/c-Met binding. IL-1 and Prostaglandin E2 (PGE2) are also involved in β-catenin nuclear localization and transactivation, according to an intensive cross-talk between CRC cells and MSC, as demonstrated by Li *et al*.^[54]^. Indeed, CRC-derived IL-1 binds its receptor on MSC surface and induces PGE2 production; MSC-derived PGE2 hyperactivates Akt signaling, thereby leading to β-catenin nuclear translocation and inducing stemness properties of CRC cell lines, such as EMT and invasion. Furthermore, the release of IL-1 in microenvironment induces MSC to produce other specific cytokines and chemokines, such as IL-8 and IL-6, involved in affecting the stemness of CRC cells^[54]^.

**Table 1:** Soluble factors involved in stemness. All types of cells in TME release specific soluble factors involved in stemness pathway promotion

TME cells	Soluble factors	Target cells	Signaling pathway	Biological effects	Ref.
Myofibroblasts	HGF	CSC	Wnt/β-catenin	Clonogenicity	[53]
MSC	PGE2	CRC	Wnt/β-catenin	EMT and invasion	[54]
Endothelial cells	JAG1	CRC	Notch	CD133 expression, tumorigenicity and chemoresistance	[55]
CAF	IL-17A	CIC	Wnt/β-catenin	Chemoresistance	[56]
CAF	HGF/SDF1	CSC	Wnt/β-catenin	CD44v6 expression, undifferentiated status and clonogenic activity	[27]
CD4^+^	IL-22	CRC	STAT3/DOT1L	Regulation of stemness genes	[58]

HGF: hepatocyte growth factor; CSC: cancer stem cells; MSC: mesenchymal stem cells; PGE2: prostaglandin E2; CRC: colorectal cancer; EMT: epithelial-mesenchymal transition; JAG: jagged; CAF: cancer-associated fibroblasts; IL: interleukin; CIC: cancer initiate cells; STAT: signal transducer and activator of transcription; DOT1L: disruptor of telomeric silencing 1-like

Another actor in conferring stemness properties to CRC cells is JAG1, released by endothelial cells: without direct cell-to-cell contact, endothelial cells produce JAG1, which activates Notch signaling in CRC cells. More specifically, JAG1 modulates stem cell-like features including increased CD133 expression, CRC cell sphere-forming capability, enhanced tumorigenicity and oxaliplatin-resistance^[55]^. At the same time, it has been demonstrated that also the cytokine IL-17A is involved in resistance to chemotherapeutic agents: chemotherapy-treated cancer associated fibroblasts (CAF) enhance cancer initiate cells (CIC) growth through IL-17A secretion; indeed, IL-17A binds its cognate receptor IL-17RA on CIC membrane and induces nuclear β-catenin localization and chemotherapy-resistance^[56]^. Furthermore, Todaro *et al*.^[27]^ showed that TME is able to reprogram CD44v6^-^ CSC into metastatic CD44v6^+^ cells, through CAF-released HGF and SDF1, thereby promoting β-catenin signaling, undifferentiated status and clonogenicity capabilities.

Immune cells, such as tumor macrophages, T-regulatory and -effector cells, represent a further relevant cellular population of TME and are able to influence self-renewal of CRCSC^[57]^. Kryczek *et al*.^[58]^ demonstrated that CD4^+^ T cells define CRC stemness, by releasing IL-22 and activating the transcription factor signal transducer and activator of transcription (STAT)3, involved in histone modifications of specific cluster of genes, and the histone-3-lysine-79 methyltransferase disruptor of telomeric silencing 1-like, involved in core stem cell genes like *Nanog* and *Sox2*.

## IL-8 involvement in CRC stem cells

**Figure 1:**
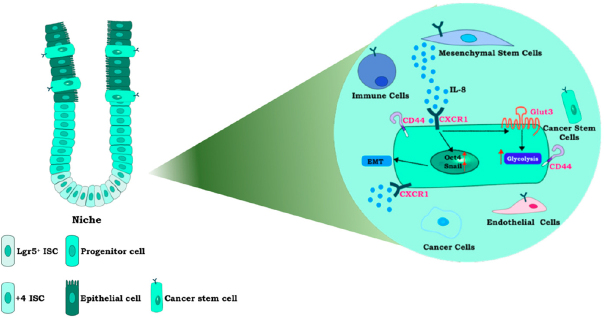
IL-8 plasticity in regulating stemness and TSI. In intestinal niche, MSC interact with CRC cells and increase IL-8 levels in TME. By the binding to CXCR1, IL-8 promotes stemness induction and maintenance through several mechanisms such as: Oct4-/SNAIL-dependent EMT, Glut3 and glycolysis induction and CD44 upregulation. CXCR1 is expressed in different cell types (i.e., endothelial and immune cells), thereby highlighting the complex role of IL-8 in regulating TSI. ISC: intestinal stem cells; EMT; epithelial-mesenchymal transition; GLUT: GLUcose transporter; IL: interleukin

As extensively reviewed by our and other groups, cytokine network represents a pivotal aspect in TME and TSI: amid the plethora of soluble factors involved in CRC progression and drug resistance, IL-8 is now recognized as one of the major promoters of tumor progression^[10]^. Several types of cells (i.e., macrophages, MSC, endothelial, epithelial and cancer cells) release IL-8 in TME: due the presence of its receptors, even in cells other than those which released IL-8, this chemokine is involved in promoting many features of cancer development, such as EMT, angiogenesis, tumor growth, metastasis and immunosuppressive microenvironment^[59]^. Indeed, through both autocrine and paracrine mechanisms, IL-8 plays pleiotropic roles in promoting specific signaling pathway activation (e.g., Snail, Slug, Akt and MAPK cascade) and inducing macrophages-derived paracrine factors production {e.g., TGF-β, EGF, IL-6, IL-1β and matrix metallopeptidases [(MMP)-2/-9]}^[60]^. Through the regulation of all these processes, IL-8 represents a novel and important cross-link between tumor inflammation and stemness in CRC microenvironment [Figure 1].

As we previously mentioned, MSC are involved in the formation of tumor stroma, by the secretion of specific paracrine factors, such as IL-8. Wang *et al*.^[61]^ demonstrated that, following the interactions with CRC cells, MSC increase IL-8 release and MSC-derived IL-8 levels are higher than CRC cells-derived IL-8 levels. MSC-secreted IL-8 is involved in paracrine-induced angiogenesis, by promoting endothelial cells proliferation and tube formation, also in nude mice models.

In addition to the well-established role in angiogenesis, one of most accredited functions of IL-8 is to affect stemness properties through the induction of Snail-mediated EMT process: Hwang *et al*.^[62]^ demonstrated that Snail-IL-8 axis regulates the stemness properties of the CD44^+^ subpopulation in CRC. In their work, the authors analyzed the transcriptomic profile of 16 primary CRC-derived colonospheres, which are mainly characterized for high expression of Snail, IL-8, VEGF and low occurrence of E-cadherin. Results show a significant correlation between Snail and both IL-8 and CD44 expression; moreover, the double Snail^+^/IL-8^+^ population significantly co-occurs in CD44^+^ cells, which display more malignant features, due to EMT activation, as compared to CD44-negative. They further identified 10 putative Snail-binding sites in proximal promoter region of *IL-8* gene, revealing that Snail directly regulates IL-8 transcription, without the activation of a IL-8 feedback loop on Snail expression. Snail-induced IL-8 roles in promoting stem-like properties were also confirmed by using shRNA or neutralizing antibody (nAb) against IL-8: with all these strategies, Hwang *et al*.^[62]^ showed a decrease of stemness genes expression and chemoresistance.

Very recently, the same group demonstrated that CRC patients, who expressed the specific CRCSC activation pattern Snail^+^/IL-8^+^, display increased MyeloPerOxidase (MPO)^+^ neutrophils which correlate with poor patient survival^[63]^. According to these data, Roncucci *et al*.^[64]^ showed that patients with CRC display higher number of MPO^+^ cells in normal mucosa rather than controls, and that MPO^+^ levels increase during carcinogenesis. Moreover, the authors correlated microsatellite stability of CRC with TME features: indeed, MPO^high^ cells are more detectable in MSI tumors as compared to MSS. Nevertheless, the role of MPO expression in CRC is still controversial, as reported by Droeser *et al*.^[65]^, who showed that MPO-positive cells infiltration is a favorable prognostic factor in CRC.

In 2018, Luo *et al*.^[66]^ also investigated the *IL-8* gene transcription as modulator of CSC-like features in CRC. More specifically, they demonstrated that mono (2-ethylhexyl) phthalate treatment increases the population of CSC and promotes the association of β-catenin-TCF complex to IL-8 promoter, both in *in vitro* cell lines and in mice.

Another stemness gene involved in IL-8 upregulation is Oct4, as demonstrated by Chang *et al*.^[67]^. The authors showed that Oct4-overexpressing CRC cells display stemness properties, such as sphere and cell colony formation, cell migration and chemotherapy resistance, as compared to parental cells; consistently, gene expression profile of Oct4^high^ cells highlight the upregulation of stemness proteins (i.e., CD133, CD44, Snail, Sox2 and Nanog), IL-8 and IL-32, which both promote CRC progression in an autocrine fashion. Furthermore, it has been demonstrated that Oct4^high^ cells-released IL-8 and IL-32 induce also tumor progression of parental CRC, by promoting stemness properties. Even in these sets of experimental data, CRC stemness features can be attenuated by using IL-8 and IL-22 nAb, alone or in combination^[67]^.

The involvement of IL-8/CXCR1 axis in expansion of CRCSC and tumor growth was also well investigated by Carpentino *et al*.^[68]^. Indeed, they first detected high levels of IL-8 in TME and a tumor volume decrease in xenografts, after IL-8 blockade. In a subsequent and very recent paper, the authors demonstrated that *in vitro* CRCSC respond to IL-8-dependent proliferation in a dose-dependent manner and knock down either IL-8 or CXCR1 results in dysregulation of cell cycle progression (cyclin D1 and B1), consequent proliferation arrest and angiogenesis inhibition. Similar results were also obtained in *in vivo* xenograft mice^[69]^.

During the time, it has been shown that also alterations of glucose metabolism are involved in stemness and cancer progression, as represented by the well-known Warburg effect^[70]^. In that respect, Shimizu and Tanaka^[71]^ demonstrated that in CRC, the glucose uptake is induced by IL-8, thus increasing CSC-like characteristics. Indeed, they identified the glucose transporter 3 as a new IL-8 target gene and that IL-8 induces the expression of glucosamine fructose-6-phosphate aminotransferase: all the mechanisms promote *O*-GlcNAcylation. Furthermore, the *O*-GlcNAcylation inhibitor OSMI1 reduces the subpopulation of CSC, through downregulation of Sox2 mRNA and protein. *O*-GlcNAcylation increases EMT and thus the metastatic capabilities of CRC: indeed, lymph node metastasis potential and low OS are observed in CRC patients with high levels of *O*-GlcNAcylation^[72]^. This evidence highlights the IL-8 potential implication in CRC treatment, as key regulator of *O*-GlcNAcylation in CSC.

The disorder of lipid metabolism is another altered metabolism involved in many aspects of CRC progression, mainly due to the activities of bile acids (BA)^[73]^. BA are cholesterol derivatives synthesized in the liver and following the conjugation with glycine or taurine, they are exported via bile in the intestine, where they regulate digestion and absorption^[74]^. Acting as signaling molecules, BA are also potent CRC promoters: indeed, BA signal through different signaling cascades, such as MAPK, PI3K and NF-κB, in order to affect transcription of several stemness genes, including IL-8^[75]^. Indeed, Nguyen *et al*.^[76]^ demonstrated that secondary BA lithocholic acid (LCA) induces IL-8 expression, by enhancing ERK1/2 activity and blocking STAT3 phosphorylation: LCA-induced IL-8 expression stimulates endothelial cells proliferation and tube-like formation, in *in vitro* models.

## Conclusion

CSC and TSI, through both cellular and soluble factors such as cytokines, orchestrate several mechanisms involved in CRC drug resistance. In CRC, IL-8 exerts its effects in regulating tumor progression, pharmacological response and stemness induction, through both intrinsic (*vs*. tumor) and extrinsic (*vs*. microenvironment) processes. In order to overcome tumor drug resistance, further IL-8-CXCR1 axis investigation could improve the development of new drugs, which can be used alone or in combination with other therapeutic agents.

## Declarations

### Copyright

© The Author(s) 2019.
